# Book Reviews

**Published:** 1820-03

**Authors:** 


### CRITICAL ANALYSIS

OF

### RECENT PUBLICATIONS, IN THE DIFFERENT BRANCHES OF MEDICINE AND SURGERY;

### SELECT MEMOIRS, AND HISTORIES OF CASES;

### In the Literature of Foreign Nations.

Πατρίδες ἄρα
Ανδράσιν, ἡ πατραῖς ἄνδρες, ἀγαλλόμεθα.

*Medico-Chirurgical Annuary of the Civil Hospitals and Infirmaries of Paris, or a Collection of Memoirs and Observations by the Physicians and Surgeons of those Establishments.* 4to. pp. 636, with fourteen plates in large folio. Crochard, Paris. 1819.*

FROM an advertisement prefixed to this work, it appears, that it is produced in consequence of a decree of the "conseil général d'administration des hopitaux et hospices civils" of Paris, that it should itself take the charge of the publication of an annual collection of Memoirs by the physicians and surgeons of those establishments, deduced from observations occurring to them in their public official capacity. We cannot yet give our readers any idea of the manner in which the expectations so excellent a plan for the improvement of practical medicine must excite, have been realized, because we have hitherto perused only the first memoir comprised in this collection, which is on—

*Fracture of the inferior Extremity of the Fibula, the Luxations, and the Accidents which are the consequences of it.* By M. DUPUYTREN, Chief Surgeon to the Hotel Dieu, &c. &c.

The principal object of this memoir is to show the advantages of a new mode of treating those accidents, which the author has employed to a great extent during the last thirteen years. With the intention of proving his title to originality of method in this respect, and of rendering his observations on the nature of the injury, and the principles for its treatment, perfectly clear and intelligible to every class of readers, M. Dupuytren comprises in his dissertation a historical account of the opinions which have been entertained respecting the points above mentioned, and a series of anatomical demonstrations of the structure of the foot and the mechanism of the ankle-joint. These we pass over, in order that the space we can devote to this article may comprise what may be novel to the greater part of our readers.

We commence with the experiments made by M. Dupuytren, on the dead body, to ascertain the influence of the various modes of violence

* *Annuaire Medico-Chirurgical des Hôpitaux et Hospices Civils de Paris, ou receuil de Memoires et Observations par les Medecins et Chirurgiens de ces Etablissemens, &c. &c.*

exerted on the mechanism of the ankle-joint; and on this occasion we shall combine what may be generally known with what is not so, because we consider it right that a complete account of the results of those experiments should be here given.

Force applied to the foot, so as to produce either inordinate flexion or extension of it with respect to the leg, produces, according to its degree, distension, rupture of the lateral ligaments of the joint, or separation of a portion of the structure of the malleoli; but it does not, in the dead body, produce luxation of the joint either forwards or backwards. A slight effort to bend the foot sideways, either inwardly or outwardly, beyond the ordinary extent of that motion, produces only simple distensions of the ligaments, which represent what are termed *strains*. A greater effort of this kind produces separation of the ligaments from the malleoli, by tearing away some of the compact tissue of those bony processes, or a separation of their periosteum, without the ligaments suffering the slightest solution of continuity; a thing which very often happens, too, in the living body. A more rapid and violent effort produces, not rupture of the ligaments, as might be supposed, but that of the malleoli themselves. In the case of violent bending of the foot outwardly, separation of the internal lateral ligaments, or rupture of the corresponding malleolus, always precedes the fracture of the fibula. In a similar motion of the foot inwardly, the fibula is almost always fractured, whilst the internal malleolus and lateral ligaments remain without lesion. In both these cases, the malleoli are fractured by traction of their extremity, and this traction becomes more efficacious in proportion as the lateral ligaments, changing their direction by the deviation of the foot, more approach the perpendicular direction with respect to the malleoli. We see, however, the lower part of the fibula fractured from a violent bending of the foot outwardly, or the foot being fixed on a flat surface, on the limb and weight of the body taking the same direction with respect to the foot: still this fracture has always been preceded by rupture of the internal lateral ligaments, or fracture of the corresponding malleolus, by traction; and then the fibula becomes fractured, from the great pressure of the foot, now turned outwardly, on the external malleolus; or from the weight of the body, its axis being altered, falling obliquely on the extremity of the fibula. The fracture of this bone, in this case, takes place ordinarily within the distance of a few inches from its lower extremity.

If, with the foregoing observations, the action of the muscles be taken into consideration, correct ideas will readily be formed of the nature of strains, fractures of the lower extremities of the bones of the leg, and the consequences of the latter accidents.

Fracture of the fibula arising from violence immediately applied to some part of its course, is not accompanied with any distortion of the foot when the fracture occurs about the middle part of that bone, or not within a short distance of the malleolus; but, when the latter takes place, the action of the muscles will sometimes draw the foot out of its proper direction, which is commonly outwardly. In the former case, the muscles of the leg, and the attachment of the fibula to the tibia, prevent sufficient displacement of the former bone to permit the foot to become distorted: in the latter, those supports being wanted, some degree of distortion of the foot will take place, but it is not equally great with that occurring when the internal ligaments of the joint are separated from the corresponding malleolus.

The occasion and consequences of fracture of the lower extremity of the fibula from *indirect* causes, or those acting on the mechanism of the ankle-joint, will be readily understood from the account of the results of the experiments above alluded to, and attention to the influence the muscles will have on the foot during or after the occurrence of the fracture. But, some illustrations of those principles by a few cases, may be of utility to some of our readers.

A man running along a room, placed his left foot on a bone lying on the floor, and at the same instant turned suddenly round towards a noise he heard behind him: his left foot slipped from under his body, which fell to the ground on the left side, with the limb bent and lying on its external surface. On being brought to the hospital, his foot was luxated, and turned outwardly, and there was fracture of the fibula at two inches distance from the external malleolus. The foot could be readily brought into a straight direction with respect to the leg, but, on being left to itself, it again turned outwardly. Here, then, was fracture of the fibula by traction on its extremity; but the foot was not turned inwardly, as it would remain in the dead body: the muscles drew it outwardly. Those who have not forgotten their anatomical instruction, will know those muscles to have been the abductors, the chief of which are the peronei posticus and tertius; but their superiority over the adductors in this case arises from the change in the situation of the pulley they now act on; because, in children, whose ligaments are weak and lax, we find it is the adductors which always have the superiority, and their feet are turned inwards.

This case, then, of fracture of the fibula from traction on its extremity, is the most simple of the two, because the injury of the joint is less than what occurs in the following species:

A man sprung out of the right side of a cart, from the horse in it being ungovernable and running at full speed: he came on the ground on his feet, just before the wheel. The horse at this instant turning suddenly round, he hastily retracted his left leg, in the act of retreating, and then fell to the ground on the internal side of the right leg. He felt at the instant of the fall violent pain, both in the situation of the internal malleolus and on the outer and lower part of the leg; the foot was turned outwardly. The patient had made no effort to raise himself from the ground. Here, then, lesion of the internal malleolus first took place, and the fracture of the fibula was the consequence of the weight of the falling body being thrown obliquely on the extremity of that bone. On his being brought to the hospital, the foot was turned outwardly quite up on the leg. The lower internal extremity of the tibia made an angle, the skin over which was divided transversely, disorganized, showing a large spot of ecchymosis; beneath which the fragments of the internal malleolus could be felt, fractured transversely and drawn downwards by the internal ligaments of the foot.

Those two are common accidents; the following is of rare occurrence:

A woman was thrown down in the street by a light one-horse chaise, the wheel of which passed over the ankle of the left leg, as this lay on the ground on its outer surface. Great contusion was manifest at the basis of the inner malleolus, but there was no apparent deformity. The patient, notwithstanding the severe pain she felt, attempted to walk, supported on each side by the arms of two persons: she had made but a few steps, when she heard a crack, and felt severe pain a little above the outer malleolus, and the foot turned outwardly; but, being supported, she did not fall to the ground. On examination, now, there was ecchymosis at the inner and lower part of the leg, just about the ankle, crepitation at the basis of the internal malleolus, the foot was turned outwardly, and there were signs of fracture of the lower extremity of the fibula. Here it appears that the internal malleolus was broken off by the wheel of the chaise; the corresponding ligaments then failing to support the foot on this side, permitted the axis of the body to change, and its weight to fall obliquely on the extremity of the outer malleolus; the consequence of which was fracture of the fibula.

It is but rarely that so precise a history of the accident can be obtained as in the foregoing cases; but the second species may in general be distinguished from the first, by the greater degree of distortion of the foot, and generally by ecchymosis, or more violent injury, of the soft parts about the inner malleolus. The diagnosis is not of much importance, because the mode of cure should be similar in both cases; only varied in energy and duration proportionate to the greater violence of one than of the other.

M. Dupuytren treats of fracture of the fibula as it is simple, or complicated with lesion of some of the parts constituting the ankle-joint. Of each of those species he makes several varieties. Those of the first are, 1°, fracture at more than three inches distance from the summit of the malleolus externus; 2°, fracture at less than three inches from the summit of the malleolus externus. In the former no distortion of the foot can happen as a consequence: in the latter, in whatever way arising, that is, whether mediate or immediate, distortion of the foot *may happen*. When it arises mediately, or from violence applied through the mechanism of the ankle-joint, displacement is generally a simultaneous and persistent consequence; but the same thing may also happen when the fracture arises from violence immediately applied to the bone after an indeterminate interval, especially if the patient attempts to walk on the foot.

The varieties of the complicated species consist in the presence of one or more of the following circumstances with the fracture of the fibula: rupture of the lateral ligaments, of the malleolus internus, or of the tibia itself; displacements of the foot outwardly, backwards, and sometimes inwardly;* ascension of the astragalus and the foot

* We call displacement of the foot *inwardly*, that distortion in which the sole of the foot is turned inwardly and more or less upwards; but M. Dupuytren calls along the external surface of the tibia; effusion of blood; rupture of the integuments; projection of the bones through the skin; swelling; tension; inflammation; gangrene; suppuration; necrosis; delirium; and diverse forms of fever.

We shall only consider in a particular manner such of the above accidents as present circumstances of considerable importance, joined with what may be novel to many of our readers: the rest we leave to their own reflection.

Fracture of the inferior extremity of the tibia sometimes happens, instead of separation of the lateral ligaments from the internal malleolus or fracture of this bone. This almost always takes place consequently to that of the fibula, and as an effect of the causes which have occasioned the latter accident. In the account M. Dupuytren gives of this, he is not consonant with what he stated in a foregoing part of his memoir: he says, "When the fibula has been fractured by violent abduction of the foot, the effort producing which motion is continued on the tibia, and the lower extremity of the latter bone finds a support on the ground: then, instead of acting on the internal ligaments or malleolus, the force is wholly transferred to the tibia, which becomes fractured at a short distance from its inferior extremity." He had before asserted absolutely, that "separation of the internal lateral ligaments, or rupture of the corresponding malleolus, always precedes the fracture of the fibula," from violence of this kind. We must leave this point to our readers; for our own observations and literary researches have not furnished us with any facts calculated to settle the difference here indicated. It is however probable, that the accident under consideration may occur in the manner above described; and the only remarkable thing may be, that M. Dupuytren is not consonant in his statements. Sometimes fracture of the tibia will precede that of the fibula, and the latter may occur either from the same cause or as a consequence of the former, in the same manner as it did in the patient whose leg was overrun by the wheel of the chaise above related.

The fracture of the tibia is here generally oblique; and hence, according to the direction in which the bones ride over each other, the foot may be turned either inwardly or outwardly, or project backwards or forwards.

Sometimes the solens and gemelli muscles draw the foot backwards: this takes place to a great extent only when there is lesion of the inner malleolus, and then the foot is also turned outwards. Sometimes there is here great difficulty in keeping the foot in the proper position.

The turning inwardly of the foot is of very rare occurrence: even in cases of fracture of the lower extremity of the tibia without that of the fibula, the foot is almost always turned a little outwardly. The cause of the extraordinary accident above mentioned cannot be satisfactorily explained: it only occurs when the tibia is fractured, and

---

this, displacement *outwardly*, because the *astragalus is turned outwardly*. We think the old mode of designating the accident is the most familiar, and therefore adhere to it, as we did in the foregoing part of this article.

probably arises from some peculiarity in the direction of this, and in the resistance made by the lower part of the fibula.

Necrosis, or death of the parts, but rarely occurs in the separated portions of the bones; but it not unfrequently happens to the tendons, and generally in those of the peroneal muscles, from their rubbing against rough or displaced portions of bone. In this case, after a longer or shorter interval from the accident, the integuments over the affected parts become inflamed, suppurate, open, and fibrous filaments pass out, until all that have been destroyed escape; cicatrization then begins, and is soon completed: but what remain of the tendons, become weak and adherent to the skin, admit of only difficult, confined, and sometimes painful, motions.

The mode of treating fracture of the fibula with luxation of the foot, in general use in England, is that of Pott; but, with the greatest care and attention, some degree of distortion of the foot, or confinement of its movements from a little displacement of the fractured portions of the fibula, has been a frequent consequence of this accident, when thus managed. That of M. Dupuytren is obviously better in theory; and practice seems to have sufficiently established its advantages. Mr. Charles Bell has given a recent and excellent history of the mechanism of the various ways in which this accident takes place; but his mode of treating it does not differ essentially from that directed by Pott: it does not appear to be calculated to prevent the falling-in of the lower extremity of the fibula towards the tibia, which is the cause of the general ill consequences of this accident. When the foot is brought into the state of extreme adduction, in a case of fracture of the lower end of the fibula, it exerts, by means of the lateral ligaments, a traction of the summit of the outer malleolus in an inward direction, and consequently the fractured portion corresponding to it is drawn outwards from the tibia. It is on this principle that M. Dupuytren's mode of treating this accident is founded.

Fracture of the fibula, then, which is accompanied with luxation of the astragalus inwards, (that is, with distortion of the foot outwards,) requires, in all cases, an apparatus which maintains the foot turned inwards, and the inferior fragment of the fibula raised from the tibia, and in the direction of the superior fragment.

If, with the advantage of fulfilling the above indications, M. Dupuytren continues, this apparatus join those of being sufficiently simple in its mechanism that it may be understood by persons of the least intelligence; of being made of matters so common, that they may be every where found; of being applied without difficulty by persons of the smallest experience; this apparatus will comprise all the conditions which ensure a durable success in surgical instruments,—efficacy united to simplicity. Such appears to be the case with the following apparatus:

A cushion, a splint, and two bandages, entirely compose it: the cushion made of linen, and stuffed two-thirds full of hair-balls, or chaff enclosed in bags, in the usual manner, should be two feet and a half in length, four or five inches in breadth, and three or four inches in thickness. The splint, from twenty to twenty-three inches in length, three inches in breadth, and nearly half-an-inch in thickness, should be made of firm, or but little flexible, wood. One of those ordinarily employed in fractures of the leg might serve for this use. Lastly, the two bandages, made of linen a little worn, should be from four to five yards long.

The cushion, doubled in the form of a wedge, should be applied on the internal side of the fractured limb, its base below, and resting on the internal malleolus without passing beyond it; its summit above, on the internal condyle of the tibia, so that it protects the limb from the splint; it furnishes to this a support, which sustains it at the distance of several inches from the internal margin of the foot; and, lastly, it serves to throw the tibia outwardly.

The splint, applied along the cushion, should extend six or seven inches below it, and which will be about four inches below the internal margin of the foot.

These two parts of the apparatus, thus disposed, should then be fixed by one of the bandages passed round the limb below the knee, when the portion of the splint prolonged below the cushion will leave between itself and the foot an interval of several inches, and furnish a *point d'appui* to which the foot may be drawn, from without inwardly. In order to effect this, the second bandage should be drawn from this point over the instep and the heel, alternately embracing the splint and the parts of the limb just indicated in circles, gradually lessening, and forming the figure of the cypher 8 with the crossing part on the splint. Thus, the apparatus acts on the principle of a lever of the *first kind*, in which the *point d'appui* is the base of the cushion a little above the malleolus internus, and in which the resistance as well as the power acting on the fracture, are in the extremities of the foot. Thus drawn, then, it is evident that the foot must cede to the action of the lower bandage, aided by the elasticity of the splint, both which tend to draw it inwards; and that, in proportion as the foot cedes to this double power, the tibia, pressed by the base of the wedge formed by the cushion, and on which the whole apparatus has its support, must be driven outwards, as well as the astragalus. Lastly, it is evident that the lower fragment of the fibula, drawn downwards by the external lateral ligaments of the ankle-joint, must exert on the external surface of the astragalus a tilting movement contrary to that which displaced it, and by which it was drawn from its natural situation.

We must not confine ourselves, if we would obtain a complete reduction, to drawing the foot in a perpendicular line under the limb; it must be brought as much inwards as it had been turned outwards by the peroneal muscles; that is the principle of the treatment: its execution is not attended with difficulty, and is not subject to any inconvenience; it does not even produce pain. It may happen, that the foot, after having been retained during the whole treatment in a state of forced adduction, will not return to its ordinary position immediately after the apparatus is removed: this inconvenience is, however, so slight, that it would not merit being noticed, were there not a mean of causing it to disappear in a few days; which is, the application of the same apparatus to the outer surface of the leg and foot.

When the foot is drawn backward and upward, the mode of treatment of M. Dupuytren is similar to that in use amongst us. The same cushion and splint used for the former variety of fracture, are to be applied to the back part of the leg in the same manner; only here the cushion must extend below the heel. The first bandage is to be applied below the knee, the second round the lower end of the tibia and the splint: this is the acting part of the apparatus. The turns of the bandage, acting on the splint and the tibia, force the heel forward and the tibia backwards; and this power may be applied with sufficient effect to preserve the astragalus in its proper situation. It is prudent to place a square pad between the lower bandage and the tibia. The duration of the treatment in complicated cases, was usually from twenty-five to thirty days.

Of two hundred and seven cases of fracture of the fibula, comprising all the varieties of this accident, treated in this way by M. Dupuytren, two hundred and two were cured; the remaining five died: three of them from the consequences of the injury itself; two from complications independant of the injury. Amputation was never performed, either primitively or secondarily.

Seven-tenths of them affected the right leg; six-tenths arose as a result of violent adductive motions of the foot; three-tenths from similar abductive movements; one-tenth as a consequence of blows, or the passing of some heavy body over the external and inferior part of the limb.

With respect to the seat of the fracture of the fibula: in five-tenths it existed at two inches from the summit of the external malleolus; three-tenths below this point; two-tenths above the same point. Those which had their seat within two inches from the summit of the external malleolus, were often complicated with displacement of the foot; the others but rarely presented this occurrence.

Seven patients had necrosis of some of the affected parts. In four instances it affected the bones; in three of which the internal malleolus, or inferior extremity of the tibia, was the seat of it: the extremity of the fibula in only one instance. The first "arose from suppuration, which deprived the bones of the fibrous parts which enveloped them; the last was the result of primitive and complete separation of scales from the body of the fibula. In all, thickening and ossification of the periosteum replaced the loss of the bony parts which had been removed."

The three other cases of necrosis affected the tendons: two occurred in the tendons of the tibialis anticus and the extensor longus pollicis pedis, and were produced by the projection forwards of the superior part of the fracture of the *tibia*, (M. Dupuytren says,) itself caused by the displacement of the foot backwards; one only affected the tendons of the peronei posticus and tertius, and was a consequence of laceration and other injury which those tendons had suffered from the fractured part of the superior fragment of the fibula. These necroses retarded the cure, but they did not in any instance sensibly interfere with the motions of the parts.

*On Mediate Auscultation*, &c. By R. T. H. Laennec, M.D. &c.

On resuming the consideration of this work, we commence with the author's observations respecting *pulmonary phthisis*, of which, he says, the existence of tubercles is the cause and proper anatomical character: ulceration affecting the bronchial surfaces of idiopathic origin and without the existence of tubercles, is not considered to form a variety of phthisis. As this is merely a nosological question, we shall leave it, to direct our attention to the author's observations on tubercular phthisis. There is but very little that is novel in M. Laennec's pathology on this occasion; yet, we shall give a general view of it, for the sake of convenience of reference, when we come to notice his new means of diagnosis.

Tubercles are developed under the form of small semi-transparent grains of a grey hue, but sometimes diaphanous and almost colourless. Their size, in the first instance, varies from that of a millet to that of a hemp seed; they at length become larger, opake or yellowish, at first in the centre, and successively throughout their extent. Those most adjacent unite as they develope themselves, and form then more or less voluminous masses, of a pale yellowish colour, opake, and of a density similar to that of the hardest sort of cheese: they are then termed *crude tubercles*. It is ordinarily towards this epoch of the disease, that the tissue of the lungs, hitherto healthy, begins to become hard, greyish, and semi-transparent around the tubercles, from a new production of tuberculous matter in the first stage of its formation, which infiltrates the pulmonary structure: the latter is sometimes found without tubercles. The pulmonary tissue thus engorged, is dense, humid, and wholly impenetrable to the air. Numerous smaller, opake, yellow spots then become dispersed in it, and at length extend throughout it.

In whatever way tubercles are formed, they terminate, after a period of various duration, by becoming soft and at length liquid. This softening begins in the centre of each mass, and gradually extends to the circumference.

Tuberculous matter at this stage is in two different states: it either resembles a thick, inodorous pus of a deeper yellow colour than the tubercles; or it is separated in two parts, one of which is very liquid, more or less transparent or colourless, unless it be tinctured by blood; the other opake, and of the consistence of soft and friable cheese. The latter M. Laennec says, is particularly found in scrofulous subjects.

When the tuberculous matter is completely softened, it opens a passage into some one of the adjacent bronchial tubes; this opening is smaller than the diameter of the cavity left after the evacuation of the matter, and remains fistulous.

Many excavations of the above kind generally co-exist, and cavities successively formed, often open into each other, forming anfractuous excavations of various forms and extent. Bands or columns of condensed pulmonary tissue, often infiltrated also by tuberculous matter, frequently traverse these excavations. Bayle, whose work on Phthisis contains the whole of the foregoing observations, stated that these bands were traversed by blood-vessels; but M. Laennec has hardly ever found a vessel of any considerable size in them. The vessels naturally existing in the pulmonary structure are obliterated in those bands. The tuberculous matter, on being developed, presses aside the proper structure of the lungs, and its blood-vessels are found, often very large in size, winding about the parietes of the cavities soon after the softening of the tuberculous matter, and forming even part of those parietes. These vessels are ordinarily flattened, they are rarely obliterated, but those of their ramifications which are directed towards the excavation or towards the tuberculous masses, are evidently so, and liquids injected into those vessels will not pass into the cavities, as Dr. Baillie long since observed.

The ramifications of the bronchiæ appear to be rather enveloped in, than pressed aside, by tuberculous matter; and the compression excited on them, apparently, promptly destroys them, for they can hardly ever be distinguished in the tuberculous masses; and yet it is very rare to find an excavation, however small, in which one or more bronchial tubes of different diameters do not open, and in a direction which makes it evident that those tubes were originally extended through the tuberculous matter.

The parietes of those excavations, in proportion as they are formed, become lined with a sort of thin, smooth, soft, and almost friable, false membrane, of nearly an opake white hue, and which is easily removed by scraping it with a scalpel. Sometimes a finer membrane of the same kind is formed in spots on the sides of the cavities; or such a membrane will exist beneath the former, but not adherent to it, and which is attached more intimately than the former to the texture which it lines. It seems to be the first degree of development of the former. Sometimes neither of these membranes is present, and the sides of the excavations are formed by the pulmonary texture, ordinarily hard, red, and infiltrated by tuberculous matter, in different degrees of development.

M. Laennec states that Bayle had thought that the false membrane above described, secreted the pus expectorated by the patient; but M. Laennec says he thinks that the greater part of the expectorated matter is the product of bronchial secretion, increased by the irritation existing in the lungs. We have not now the work of Bayle to refer to; but, unless our memory deceives in this instance in an unusual manner, he had said the same: that is, that the matter resembling pus came from the cavities, but the greatest part of that expectorated was produced from the mucous membrane of the bronchiæ.

If the disease now remain stationary, there is developed, here and there, beneath this false membrane, flakes of greyish white, and semi-diaphanous matter, of a texture analogous to cartilage, but a little softer, and which adheres intimately to the pulmonary tissue. These flakes at length increase so as to unite, completely line the ulcerous excavation, and terminate, as by continuity of substance, in the internal membrane of the bronchial tubes which open into those cavities. Sometimes this cartilaginous substance is of a light red colour, apparently from a development of very fine blood-vessels; sometimes this formation is as ancient as the tubercles themselves—an affection which constitutes the encysted tubercles of Bayle. It adheres strongly to the surrounding structure of the lungs, but the tubercles it envelopes can be separated from it, although also firmly adherent, and the internal surface of the cyst is then found smooth and polished, although unequal in its surface. M. Laennec says he has never seen those cysts, whether formed previously or subsequently to the tubercles, pass into the osseous state.

We now arrive at M. Laennec's opinions respecting the origin of those tubercles. He says that M. Bayle has perfectly demonstrated that they cannot be regarded as an effect or a termination of inflammation. We think the truth of this proposition is not demonstrated; it is at present only a probability. He advances no new arguments in support of it of any importance, but repeats those of Bayle. He objects, however, and apparently with propriety, to the specific divisions of tubercles made by the physician just named; and shews that the nearly diaphanous miliary granulations are tubercles in the first stage of their developement. The second is the state of grey tubercles, more voluminous, and yellow and opake in the centre. The third is that of entirely opake tubercles, but still of firm consistence. The fourth, that of softened tubercles, especially in the centre. The fifth, those of excavations, more or less completely empty. When they are seated in the bronchiæ, they, as well as the bronchial glands, are commonly nearly black; the cause of which was several years since shown by Dr. Pearson. It is the superior lobe of the lungs in which they are generally seated; their development at least commences here, and thence extends downwards.

Neither mediate nor immediate *auscultation* furnishes any decisive signs of the existence of tubercles during their first three stages; for when tumors of the lungs are small, and the tissue of the lungs healthy in the intervals, however numerous may be the tumors, the sthethescope does not indicate their existence. M. Laennec states that he has often heard respiration effected with an equal force and clearness in both sides in subjects which, on being opened, showed one lung healthy or containing only a few very small tubercles, and the other filled with tumors of the same kind, but of a size varying from that of a millet seed to that of a filbert; and of which the number was such, that the weight of this lung was double or triple that of the healthy one. This is applicable to other small tumors of the lungs, as well as those expressly productive of phthisis. Percussion in these cases is not attended with any diminution of sound; as soon, however, as any number of tubercles unite into a mass, respiration ceases to be heard in this part; but, as the same effect results from inflammatory engorgement and serous effusion, no indications can ever be hence derived respecting the first stages of tubercular phthisis. But as soon as the tubercles become softened, and excavations begin to be formed communicating with the bronchial tubes, the exploration of the *voice* by the sthethescope gives rise to pectoriloquism. When this is *clearly evident*, it is alone a certain pathognomonic of phthisis. When pectoriloquism is only what M. Laennec terms doubtful,—that is, when the voice appears a little acute, and is heard as it were at the bottom of the cylinder, without evidently passing through the tube, it can furnish only presumptive evidence of the existence of a deep seated excavation, or of one nearly filled with tuberculous matter, when it is heard below the fourth rib and on one side only. This sort of pectoriloquism exists sometimes in healthy persons, whose voice is very acute, between the scapula and spinal column, about the points corresponding to the origin of the bronchiæ, and on the upper part of the chest, as low down as the third rib. In meagre children, whose chest is narrow, pectoriloquism always exists about the parts corresponding to the origin of the bronchiæ. Where an excavation contains a certain quantity of tuberculous matter softened to the consistence of pus, pectoriloquism is accompanied with a sort of gurgling, which renders the sound of words indistinct. When pectoriloquism is constant and without any extraneous sound, and when no gurgling exists in the chest, it may be concluded that the excavation is entirely empty of tuberculous matter. When effusion of liquid into the cavity of the pleura exists in a point corresponding to the pulmonary excavation, the phenomenon of *egophony* will be joined with that of pectoriloquism; it should be remarked, that during the early stage of the softening of tuberculous matter, many varieties of pectoriloquism will be heard from day to day, appearance and disappearance of gurgling, egophony, cessation of pectoriloquism from matter completely obstructing the communication of the cavities in the lungs with the bronchiæ, which the attentive observer will soon be able to appreciate, but which cannot be here fully detailed.

In more than two hundred phthisical patients opened by M. Laennec, after examination during life by the sthethescope, he never once failed to find ulcerous cavities in the part of the lungs where he had perceived evident pectoriloquism. On the other hand, he has never found pulmonary excavations communicating with the bronchiæ in a subject whom he had *completely explored, and for several days in succession,* without finding pectoriloquism. It should be remarked too, that of ten phthisical patients, there is scarcely one who arrives at the fatal term of the disease, before the stage of the complete softening of part of the tubercles, and consequently of the formation of one or more excavations; it is still more rare that an excavation is formed without immediately opening into the bronchial tubes. Sometimes, even, that stage of organic lesion precedes considerably the epoch of the apparent alteration of health. M. Laennec has observed pectoriloquism in subjects who did not otherwise present any characteristic symptom of phthisis; but, on continuing to observe these patients, all the symptoms of the disease have been successively developed. Evident pectoriloquism must be carefully distinguished from simple egophony, or an erroneous diagnosis, and too unfavorable a prognosis, will be formed in several diseases different from phthisis.

Here an interesting question arises: have those cases in which pectoriloquism has been evident, uniformly terminated fatally? M. Laennec has proved the negative, and examination of several subjects after death has concurred with this circumstance, to show that patients sometimes recover apparently good health after the existence of extensive ulcerous cavities in the lungs, formed by the evacuation of softened tuberculous matter. Here then is evidence of the most decisive kind that real phthisis may be cured, and the establishment of this is one of the most interesting of the discoveries of M. Laennec. This physician has occasionally found, in persons who had been long affected with severe cough, but who had died of some other malady, anfractuous cavities in the lungs, lined by a sort of cartilaginous structure, similar to that described as being found beneath the secreting membrane of the cavities left after the destruction of the tubercles; on tracing the history of these patients, they always referred the origin of their cough to a severe disease with which they had some time previously been affected, that evinced all the phenomena of phthisis, and that too of the most severe character. In many patients who had laboured for several years under pulmonary phthisis, he has found cavities nearly, or quite empty, of tuberculous matter, and lined by a semi-cartilaginous membrane; whilst other cavities presented a lining of a softer consistence, not extended throughout the whole of the cavity, and containing a considerable quantity of tuberculous matter. M. Laennec thinks that this cartilaginous lining of the ulcerous cavities is a process by which tuberculous phthisis is sometimes cured. Many of the patients presenting the appearances just designated, had lived for several years after the disappearance of all signs of pulmonary disease, excepting the existence of pectoriloquism. Thus there are two modes of termination of pulmonary tubercles, by which the patient may escape inevitable death: in one, the cavities in the lungs become lined by a secretory membrane, like that commonly formed in fistulous sinuses in other parts; the other, by a more or less perfect cicatrization of the ulcerous cavities, by a fibro-cartilaginous structure. This structure may fill up the cavity, and, in this case, pectoriloquism will cease to be manifested. In the former the patient continues to have cough, and more or less expectoration; in the latter, he is free from these affections.

These results are, however, by no means frequent; and the subjects must be very liable to relapses, from the same disposition to tuberculous formation continuing to exist in the lungs. It should, too, be borne in mind, that similar morbid productions may frequently co-exist in other parts, as the liver, intestines, pericardium, &c. which may carry off the patient. We have seen two cases of ovarian dropsy evidently arising from the same kind of morbid formation. There were tubercles in one of the ovaries containing matter like that described above as existing in the lungs in the various stages above designated, some of which had burst and given to a watery fluid apparently secreted by the membrane lining the ovary, a greenish-yellow colour, and peculiar odour that was very remarkable. One woman* we tapped seven times; there first flowed out an almost clear and colourless watery fluid to the quantity of from fifteen to twenty pints, and then two or three pints of the

* This woman (Mrs. Engley) had been once previously tapped by Mr. Copeland, and twice before that by some person whose name we do not now recollect.

peculiar matter above described: the source of which was discovered by dissection after death. In both cases the tubercles in the ovaries varied from the size of a nut to that of an orange, and were in one, about fourteen in number; in the other twenty. In both there were tubercles in the lungs also, but which had not proceeded beyond what M. Laennec designates as the third stage in either instance.

A *vomica* of the lungs will give rise to pectoriloquism; but this can hardly ever lead to error, if the history of the case be attended to. Besides, by far the greatest proportion of cases of this kind have their origin from tubercles. M. Laennec believes that abscesses produced by inflammation of the lungs, to which the same name has been given, are very rare: he affirms, from anatomical observations, that of one hundred persons who expectorate purulent matter, this arises from tubercles in ninety-nine of them. Collections of pus in the cavity of the pleura, from inflammation of this membrane, are infinitely more frequent.

*Dilatation of the ramifications of the bronchiæ,* is a species of organic lesion that M. Laennec has occasionally met with, but which has not been noticed by any former author. This arises, he thinks, from its but rarely taking place in the whole extent of the bronchiæ, and it, hence, exists without being recognized; for, a dilated ramification exactly resembles a branch that is naturally larger in diameter, and on simply making an incision into the lungs, it might be taken for such. It is necessary, for the detection of this, to follow all the ramifications; when the large tube will be found to extend from one of a much smaller size. This dilatation is found only in subjects who have died after chronic catarrhs. It is sometimes so considerable, that ramifications which, in the natural state, would hardly admit a fine stilette, have acquired a diameter equal to that of a goose-quill, or even of a finger. The extremities of the bronchial tubes thus dilated, terminate in *culs-de-sacs* or pouches, capable of containing a grain of hemp-seed, a cherry-stone, a filberd, or even an almond. Their internal or mucous membrane, ordinarily of a red or violet colour, is evidently thickened. The cartilaginous circles constitute part of them, and appear changed into a fibrous tissue, which cannot be separated from the mucous membrane by dissection. This organic lesion may exist in all parts of the lungs; but it is most common in the superior lobe. Ordinarily it affects only a few of the bronchial ramifications; sometimes, however, it exists in all those of one lobe of the lungs. In this case, the dilatation is always greater, not only proportionally, but absolutely, in the small ramifications than in those whence they derive their origin, and in these than in their trunk. The common trunk of the bronchiæ is but rarely dilated in a sensible manner, even when its subdivisions nearly equal its diameter. When the dilatation of the bronchiæ is so extensive, the pulmonary tissue is flaccid, deprived of air, evidently compressed, and exactly in a similar state to that of a lung pressed towards the vertebral column by serous or purulent effusion in the cavity of the pleura.

When this dilatation is confined to one or two branches, no other incommodity arises from it than an habitual cough, neither very severe nor frequent, and expectoration of a little mucus: but when a great number of the bronchiæ are dilated throughout all their ramifications, there results a chronic catarrh which continues throughout the patient's life, and which even may shorten its duration, from the effusion produced by the dyspnœa, by the force and duration of the fits of coughing, and especially by the abundance of the matter spit up, which is of a greyish-yellow colour, and completely puriform. Some patients who present all these symptoms arrive at an advanced age, notwithstanding the state of infirmity which thence arises.

M. Laennec has had but one opportunity of exploring this affection with the stethoscope; and, in this case, it gave rise to imperfect pectoriloquism, as he had anticipated.

*Peripneumony,* M. Laennec has observed to present three well-marked varieties, with respect to the anatomical appearances it produces in the lungs.

In the first, the lung is heavier than natural, is of a livid or violet colour externally, and is much firmer than in the healthy state. The affected organ still, however, gives rise to crepitation on pressure between the fingers, though this is lessened in degree. When cut, its tissue appears of a livid red colour, and infiltrated with spumous, turbid, and more or less sanguineous serum, which flows in abundance from the surfaces of the incised part. The cellular and spongy texture of the lungs is still very well evident.

In the second degree, the tissue of the lung no longer crepitates on pressure by the fingers, and it has acquired a firmness and heaviness analogous to liver. The lung often appears less livid externally than in the first degree; but interiorly, it is of more or less deep red colour, and the spots of the black pulmonary matter, the ramification of the bronchiæ, the blood-vessels, and the thin cellular partitions which are irregularly dispersed in the substance of the lungs, are strongly marked. When a portion of lung in this state is cut, but very little liquid flows from the incised surfaces, and what can be pressed from them is often somewhat thick, opake, whitish, and puriform. Held between the eye and the light, the portions thus separated by incision from the rest of the lung, do not present the natural cellular appearance, but as it were a granulated structure, the grains forming it being small, red, oblong, and a little flattened. This state has been termed *hepatization.*

In the third degree, the pulmonary tissue, preserving the firmness and granulous aspect just described, becomes of a pale yellowish colour, like that of straw; and there escapes from the surface of the incised parts a more or less abundant quantity of a yellow, opake, viscous, and evidently purulent matter. This is almost the only mode of suppuration of the pulmonary tissue which arises from peripneumony. M. Laennec says, there is no organic lesion more rare than a true collection of pus, like what is termed a *vomica,* in the pulmonary tissue. Amongst several hundreds of bodies of subjects dying from peripneumony, he has only four or five times seen collections of pus in an inflamed lung.

Those three degrees of inflammation are ordinarily found combined in different ways. Very often one of the lungs is inflamed in the third degree throughout its whole extent, and the other presents only some portions inflamed in the first or the second degree. Sometimes the three exist in the same lung: the lower part is, in the generality of cases, that first attacked by inflammation, and to which it is frequently confined. Tubercles, it has already been stated, bear, with respect to their seat, the same relation to the upper lobe. This difference presents an argument of some weight against the doctrine of the essentially inflammatory origin of tubercles.

The whole of the two lungs is never found inflamed to the third, nor even to the second degree: such a state prevents respiration. But, it is not rare to find subjects in whom the whole of one lung, and more than half of the second, are totally impermeable to the air. In others, on the contrary, peripneumony produces death before the engorgement has comprised one quarter of the lungs.

Peripneumony arrived even at the third stage, may terminate by absorption of the pus, and without disorganization of the pulmonary tissue. If, in this case, the patient happens to die during convalescence, which sometimes occurs, especially in old persons, the substance of the lungs no longer presents the hepatic hardness; there is merely the density present in the first degree, or of œdema of the lung: it crepitates slightly under the finger; does not always sink in water: on being cut into, however, some very liquid pus oozes from the incised surfaces, which are of a dull yellow, or very pale greenish colour.

Chronic peripneumony, when it is not combined with the development of tubercles or of some other morbid production, presents precisely similar appearances with acute peripneumony, so that it is very difficult for a person the most accustomed to anatomical researches, to distinguish hence the character of the disease in respect to its duration.

M. Laennec depreciates considerably, we think, the value and degree of precision of the common signs of peripneumony: but a little enthusiasm is apt to mislead us all in a similar way. Mediate auscultation, will, probably, furnish decisive information of the existence of a state of vascular or cellular engorgement of the lungs; but, without the other signs, it cannot enable us to determine with certainty whether this arises from inflammation; and it is from the same signs that the most important indications, both for prognosis and therapeutics, must be drawn. One patient will die, as M. Laennec states, from inflammation affecting only one quarter of the lungs, whilst another will recover after only the same extent of the pulmonary tissue has been permeable to the air. A difference arising from the different influence such local disease has on the system in different persons.

It is from the state of respiration, that information is here derived by exploration with the cylinder.

In the first degree of peripneumony, respiration is still heard in the affected part, but it is much less sonorous than in the rest of the lungs; it is, besides, accompanied, in inspiration in particular, with a sort of crepitation, or gentle rattles, the sound of which may be compared to that made by salt when heated in a basin: these crepitant rattles are the pathognomics of the first degree of inflammation.

The second and third degrees are attended with absence of the murmur produced by respiration. Sometimes, however, more or less strongly-marked mucous rattles are heard. This takes place especially when catarrh is joined with peripneumony.

When the disease terminates favourably, the cylinder becomes a sure means of appreciating the progress of the cure. Before manual percussion indicates any diminution of the intensity of the pulmonary engorgement, the cylinder enables us to hear a slight murmur during expiration. This takes place at first only in one point, and always in the superior portion of the affected part: the sign becomes daily more strongly-marked; the part conveying it increases in extent until the state of perfect resolution. If, in the above-mentioned state, the patient makes a strong and deep inspiration, there is often heard at the moment when it terminates, a sort of crepitation analogous to that made by blowing air into a dry bladder.

On this occasion, the use of the stethoscope is of great importance; for we frequently find patients lose the pain in the chest; nearly all cough; and have only a shortness of breathing, not detected perhaps except on using exercise, or on attempting to dilate the lungs fully; who we think are convalescent; but engorgement of the lower part of the lungs still exists, and inflammation will return with violence from some occasional exciting cause.

*Gangrene of the lungs* is an affection of very rare occurrence. M. Laennec thinks it should not be regarded as a termination of inflammation from its intensity; but from some peculiarity which renders it allied to *anthrax*, &c. It is generally circumscribed, and within a small extent. M. Laennec has seen only one instance of it not circumscribed in eighteen years. The former affection is characterized at its commencement by signs of slight peripneumony, accompanied with prostration of strength and a degree of anxiety, not at all to be accounted for by the local symptoms. The sound of respiration is absent only to a small extent. As it proceeds, the patient expectorates very fetid greenish matter, and afterwards puriform sputa of a more grey colour; sometimes he suffers acute pains, and copious spitting of blood. When the disease extends beyond this period, hectic fever appears, with great heat of the surface of the body and a dull leaden colour of the skin. When excavations become formed from gangrene, pectoriloquism is developed by the cylinder. When those excavations communicate both with the bronchial tubes and the cavity of the pleura, and have produced pleurisy and pneumo-thorax, they give rise to the *metallic ringing* described in a former part of this analysis.

There is another species of gangrene which sometimes takes place in the parietes of the excavations formed in tuberculous phthisis, but this is of very rare occurrence.

